# Supplementary material for: Predicting visual function by interpreting a neuronal wiring diagram
Source: Nature. 2024 Oct 2;634(8032):113–23. doi: 10.1038/s41586-024-07953-5 (PMC11446822; doi:10.1038/s41586-024-07953-5)
Supplement: Supplementary file 6 — CRF and ERF predictions for individual TmY4 and TmY9 cells. Analogous to Supplementary Data 3, but for TmY target types. Shown are the top four monosynaptic pathways, the strongest pathway passing through each of the top ten intermediary types (ranking from Extended Data Fig. 7), and the trisynaptic pathway Tm1–TmY–Dm3–TmY (see the section entitled Prediction of spatial normalization). [file 41586_2024_7953_MOESM6_ESM.zip › DataS4/TmY9qΓèÑ/720575940627282307.pdf]

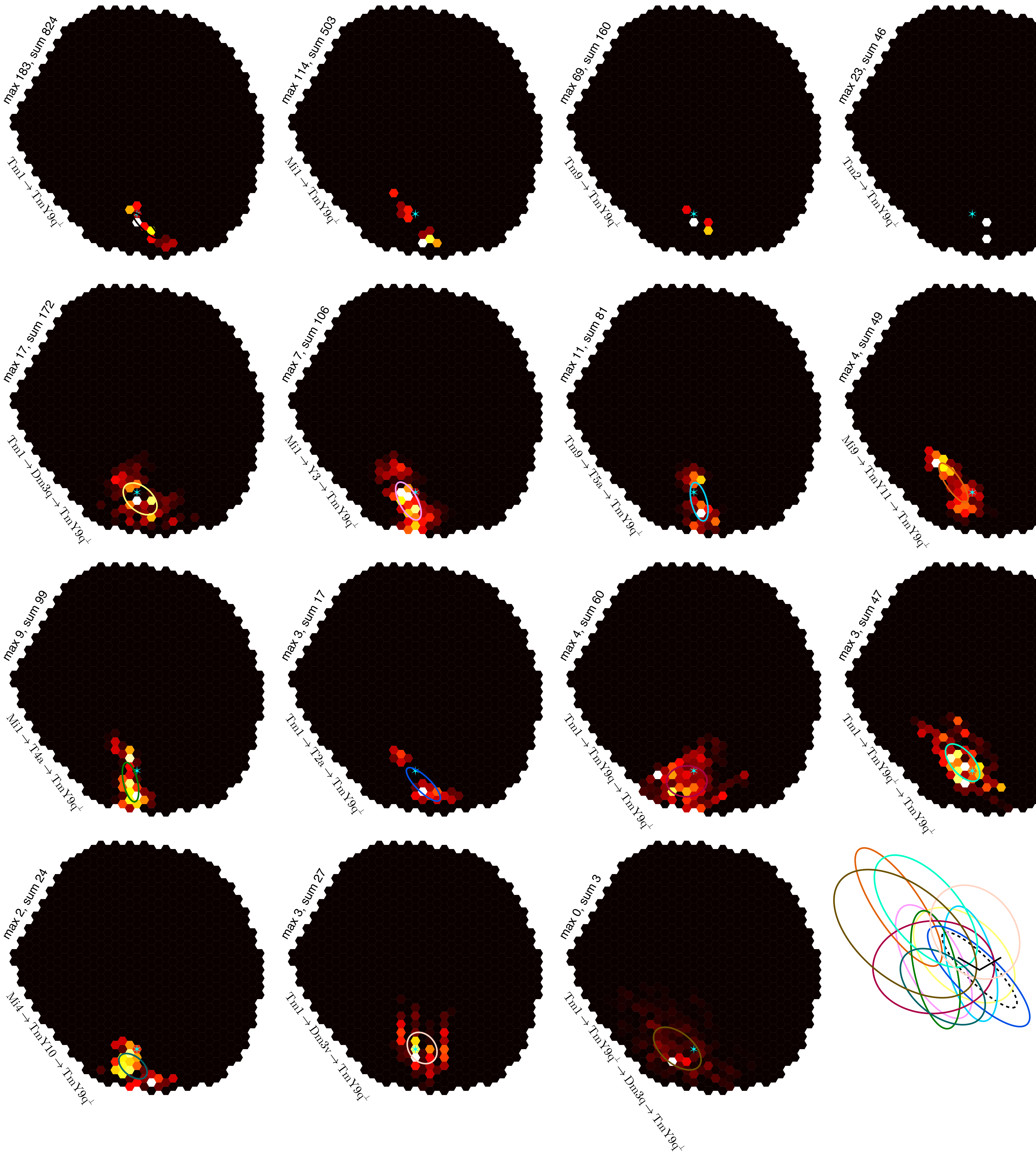
max 183, sum 824
Tm1 → TmY9q⊥
max 114, sum 503
Mi1 → TmY9q⊥
max 69, sum 160
Tm9 → TmY9q⊥
max 23, sum 46
Tm2 → TmY9q⊥
max 17, sum 172
Tm1 → Dm3q → TmY9q⊥
max 7, sum 106
Mi1 → Y3 → TmY9q⊥
max 11, sum 81
Tm9 → T5a → TmY9q⊥
max 4, sum 49
Mi9 → TmY11 → TmY9q⊥
max 9, sum 99
Mi1 → T4a → TmY9q⊥
max 3, sum 17
Tm1 → T2a → TmY9q⊥
max 4, sum 60
Tm1 → TmY9q → TmY9q⊥
max 3, sum 47
Tm1 → TmY9q⊥ → TmY9q⊥
max 2, sum 24
Mi4 → TmY10 → TmY9q⊥
max 3, sum 27
Tm1 → Dm3v → TmY9q⊥
max 0, sum 3
Tm1 → TmY9q⊥ → Dm3q → TmY9q⊥
